# It’s not me, it’s you: Anti-phage nuclease specificity inside a bacterium

**DOI:** 10.1371/journal.ppat.1013959

**Published:** 2026-02-13

**Authors:** Alex Hong, Joseph Bondy-Denomy

**Affiliations:** Department of Microbiology & Immunology, UC San Francisco, San Francisco, California, United States of America; University of Geneva: Universite de Geneve, SWITZERLAND

## Introduction: DNA-targeting systems employ diverse methods for substrate specificity

Bacteria and their viruses, bacteriophages, are the two most abundant biological entities on Earth. Their incessant reciprocal evolutionary pressure, also known as the bacteria-phage arms race, has resulted in the development of a wide breadth of diverse immune and counter-immune mechanisms [1–6]. To-date, over 150 bacterial immune systems have been discovered, a majority of which are still not well characterized [2–4,7]. CRISPR-Cas and restriction modification (RM) systems are significantly more common across sequenced bacterial genomes than any other defense system, suggesting that DNA-targeting is the most common anti-phage mechanism. In addition, more than 25% of identified defenses are predicted to have one or more DNA-binding, DNA-cleaving (nucleases), and/or DNA-unwinding (helicases) domains [8], suggesting that DNA-targeting systems make up a significant portion, if not the majority, of the bacterial defense repository. A collective international effort in discovering and characterizing anti-phage systems has revealed unique mechanisms that bacteria use to detect and restrict invading DNA. Understanding the molecular basis behind how these systems work is vital for progressing research in microbial ecology, phage therapies, and biotechnological tool development.

**Fig 1:**
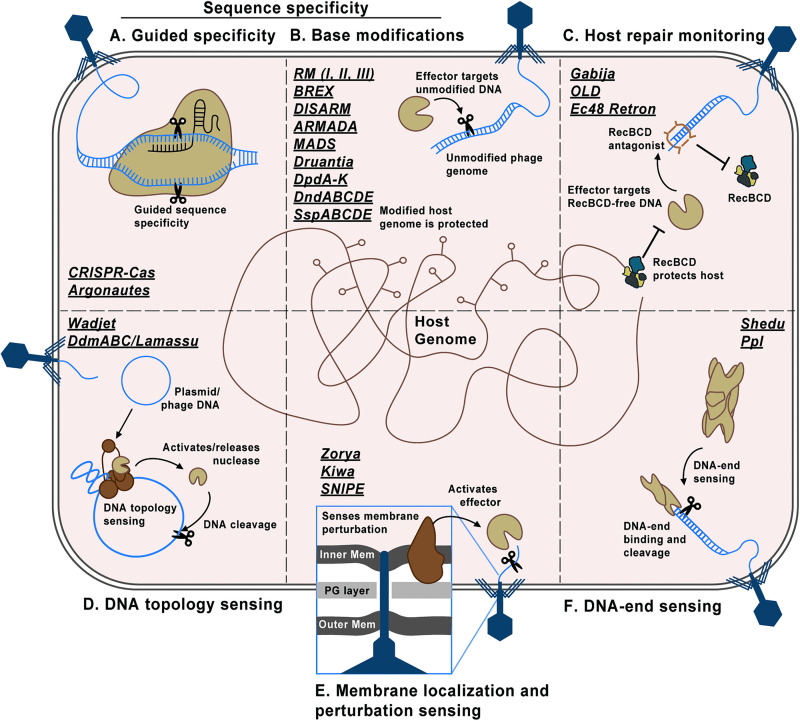
The diversity of DNA-targeting specificity mechanisms. **(A)** CRISPR-Cas (RNA-guided) and argonautes (RNA-/DNA-guided) are nuclease complexes that use complementary guide sequences to anneal and cut at target sites. **(B)** Most restriction modification (RM) and many other systems use DNA base modifications (methylation, phosphorothioation, the incorporation of 7-deazaguanine derivatives) to mark self-DNA. Unmodified DNA is cleaved. **(C)** Systems such as Gabija monitor the activity of the conserved host repair system, RecBCD. The ablation of RecBCD activity on DNA licenses Gabija activity. **(D)** The Wadjet and Lamassu families use structural maintenance of chromosomes-like (SMC-like) complexes to detect small plasmids or secondary DNA structures presumably unique to phages. Stalling of the SMC-like complexes on these substrates signals and activates nuclease effectors. **(E)** Membrane-embedded systems such as Zorya sense membrane perturbations to activate nuclease effectors local to phage-injecting genomes. Other systems such as SNIPE directly bind to phage injection machinery. **(F)** Shedu and Ppl encode nucleases  that specifically bind the ends of DNA. End-binding primes Shedu to cut at a programemd number of bases from the DNA end.

Bacteria lack a membrane-bound nucleus, and thus the bacterial nucleoid (the bacteria’s compacted genomic DNA) is not insulated from cytoplasmic nucleases. Therefore, phage DNA-targeting defenses have two options: to possess a mechanism that specifically detects and cleaves phage DNA or to achieve phage DNA destruction via indiscriminate cleavage that acts on both invader and, consequentially, host DNA [9,10]. The former (phage-specific cleavage) involves a range of distinct specificity mechanisms that underly, for example, the broad applicability and utility of systems like RM and CRISPR-Cas, the details for which we direct readers to other reviews [11–16]. Here, we summarize the field’s current understanding of how newly discovered DNA-targeting systems decide to act on invading DNA. We highlight specificity requirements for sensing and restriction, grouping systems by their use of sequence specificity and DNA modifications, DNA repair monitoring, DNA end-sensing, membrane localization, and DNA topology (Fig 1 and Table 1). We note that many other modular systems, such as CBASS, Avs, and Retrons, have large lists of effector proteins that can include DNA-targeting enzymes, but for brevity, we will focus on systems that strictly engage with DNA. We end by summarizing phage countermeasures against DNA-targeting systems to illustrate both factions of the bacteria- phage arms race. For further details on each defense system, we direct readers to the DefenseFinder wiki page [8,17].

**Table 1:** Summary of DNA-targeting systems and phage countermeasures.

*(Putative) DNA-targeting systems*	Mechanism of detectios/Specificity	Mechanism of restriction	Inhibitors/ mechanisms for evasion
***Restriction Modification (RM)***	DNA modification (methylation)	DNA cleavage	Base modification, Ocr, Ral, ArdA, ArdB, SAMase
***BREX***	DNA modification (methylation)	–	Base modification, Ocr, OrbA, SAMase
***DISARM***	ssDNA modification (methylation)	DNA cleavage	–
***MADS***	DNA modification (methylation)	–	–
***Druantia***	DNA modification (methylation)	–	Base modification, DadIII-1, Bdi1, Bdi2
***DndABCDE***	DNA modification (phosphorothioation)	DndFGH mediated dsDNA degradation	–
***SspABCDE***	ssDNA modification (phosphorothioration)	SspE mediated ssDNA degradation	–
***CRISPR-Cas systems***	Guided (RNA)	Nucleic acid cleavage	Acrs
***Prokaryotic argonauts (pAgos)***	Guided (RNA or ssDNA)	–	–
***DdmDE (a pAgo)***	Guided (ssDNA)	DNA unwinding and nicking by DdmD	–
***Nhi***	Phage SSB detection (truncated)	DNA nicking and unwinding	–
***OLD***	RecBCD inhibition	tRNA degradation	Oad1
***Gabija***	RecBCD inhibition, DNA-end sensing	Prevention of phage genome circularization, inhibition of DNA-end joining	Base modification, Gad1, Gad2
***Eco08 Retron***	Phage SSB detection	DNA cleavage	–
***Hachiman***	Phage SSB detection	DNA cleavage	Had1
***Shedu***	DNA-end sensing/binding relieves nuclease autoinhibition	DNA end cleavage	Base modification, suppression of phage replication pathway
***Kiwa***	Phage membrane perturbation and host RNAP inhibition	DNA binding	–
***Zorya***	Phage membrane perturbation	DNA cleavage	ZadI-1, Bdi1, Bdi2
***SNIPE***	Binding to phage injection machinery	DNA cleavage	–
***Wadjet***	Topology sensing	DNA cleavage	DNA topology, DNA size
***Lamassu (DdmABC)***	Topology sensing (secondary DNA stem-loop hairpins), DNA-end sensing	DNA cleavage	–
***Septu***	Phage SSB detection	DNA cleavage	–
***NixI***	Induced PLE	DNA nicking	–

– indicates unknown mechanism(s).

## Sequence specificity and DNA modifications

Sequence specificity is the most commonly known mechanism used by DNA-targeting systems to distinguish between self- and non-self-DNA, which can be broadly divided into two mechanistic classifications: RNA/DNA-guided and modification-dependent. Guided DNA-targeting systems include not only CRISPR-Cas systems, but also argonautes that are found in both eukaryotes (eAgos) and prokaryotes (pAgos) [18,19]. Unlike CRISPR-Cas, ‘long’ prokaryotic argonautes do not require PAM sites and use either single-stranded DNA or RNA guides to restrict invading plasmids or viral genomes [20,21]. pAgos also exist in ‘short’ forms, where guided detection of foreign DNA by a catalytically inactive argonaute is accompanied by the recruitment of effectors [20]. For example, the DdmE nuclease-helicase is inactive as a dimer until DdmD, a DNA-guided short pAgo, recruits DdmE to its target site on plasmids, upon which DdmE transitions to an active monomer for processive DNA degradation [22,23]. Guide acquisition has been explored in two pAgo systems, but broadly remains to be understood. *Thermus thermophilus* pAgo obtains guides through initial unguided DNA cleavage while *Alteromonas macleodii* pAgo and related pAgos employ companion HEPN RNA endonucleases [24,25]. Interestingly, guide acquisition is indiscriminate, as pAgos can acquire guides that target the host genome; in these cases, self-targeting might be avoided by a threshold requirement, where MGE infection and replication exponentially increase copies of non-self guides to overcome the autoregulatory effects of pAgo oligomerization [26,27].

The presence, or absence, of sequence-specific DNA modifications can also be used by DNA targeting systems to block or enable DNA degradation. Such systems typically include an enzyme that modifies a base in specific target sequences, which are usually found in the host genome. Unmodified invading DNA is recognized as foreign, and either cleaved by restriction enzymes (Type I, II, and III RM) or restricted by unknown mechanisms (BREX, DISARM, ARMADA, MADS, Druantia) [2,11,28–36]. Some exceptions are modification-dependent restriction endonucleases (MDREs) such as Type IV RM and END nuclease systems that act upon modified DNA [11,37]. To-date, modifications in prokaryotic defenses include methylation used by the aforementioned modification systems, phosphorothioation (PT) used by DndABCDE and SspABCDE, and 7-deazaguanine derivatives used by Dpd [38–43]. The systems with five or more subunits—DISARM, MADS, and the PT systems—likely involve additional substrate requirements for non-self detection. For example, DISARM and SspABCDE modify and/or cleave ssDNA.

## DNA recombination and repair

DNA repair is a core conserved process that maintains genome integrity. Almost every sequenced bacterial genome encodes some combination of RecA recombinase, single-stranded binding proteins (SSBs), Rec(F)OR, and one or more of the multifaceted RecBCD, AddAB, and AdnAB repair complexes [44]. Of these systems, the RecBCD complex is the most extensively studied in *Escherichia* and *Pseudomonas* [45–47]. Canonically, RecBCD loads onto the ends of dsDNA breaks. The RecB nuclease-helicase and RecD helicase subunits then translocate and unwind these ends with high processivity until the complex encounters a “Chi” sequence, where RecC recruits RecA to the newly formed 3′ ssDNA loop for recombination. Many phages encode proteins that interfere with RecBCD activity, presumably to prevent RecBCD degradation of their genomes if the phage doesn’t have many Chi sites and/or to allow phage DNA recombination/repair machinery to operate unencumbered [48,49]. Increasing evidence suggests that deviations from regular DNA repair operations can be ‘monitored’ by DNA-targeting systems to identify invading phage DNA.

DNA-targeting systems may perceive linear DNA that is devoid of RecBCD activity as an indicator for non-self-DNA. This can occur when phages inhibit RecBCD loading and/or activity. For example, in *P. aeruginosa*, Gabija prevents phage genome circularization on DNA that is devoid of RecBCD activity, following inhibition of RecBCD loading by phage end-binding proteins such as JBD30 phage’s Mu Gam homolog [50]. If DNA is a RecBCD-substrate, RecB and RecD enzymatic functions prevent Gabija from acting on DNA. Other systems such as overcoming lysogenization defect (OLD) nucleases and Retron Eco6 (Ec48) (transmembrane effector) have also been shown to become active against phages upon direct RecBCD inhibition via lambda Gam or T7 gp5.9 binding [51,52].

Alternatively, phages commonly encode proteins to assist in replication, repair, and recombination. This includes single-stranded DNA-binding and annealing proteins, which are found across all kingdoms of life. They act by binding along ssDNA to both protect from nucleases and recruit other replication or repair proteins [53]. Certain systems, including DNA-targeting Nhi, Hachiman, and Eco8 Retron (OLD effector) systems, are thought to be activated upon detecting phage SSBs [54–57]. Nhi-sensitive phage Jbug18, which has a truncated SSB, can escape Nhi—a single gene nuclease-helicase—by acquiring a full-length SSB from Nhi-resistant Andhra, suggesting that variation in phage SSBs determines Nhi activity [57]. Multiple phages were also shown to acquire SSB mutations to escape Hachiman [56]. The mechanism of SSB detection for both systems is still unknown. SSBs are also thought to bind the msDNA of Eco8 retrons, which lead to a conformational change that relieves the autoinhibition of Eco8’s OLD effector to then proceed to indiscriminate nuclease activity [54].

## DNA-end sensing

Most phages, particularly tailed phages (*Caudoviricetes*), inject their genomes linearized. This makes DNA ends an opportune substrate to attack by anti-phage nucleases. For example, Type I Shedu nuclease, EcSduA, forms a homotetrameric complex with a N-terminal clamp that specifically binds the DNA-ends [58]. DNA-end binding leads to nicking of bound DNA at a fixed distance from the ends via EcSduA’s PD-(D/E)XK nuclease domain. Furthermore, T6 phages escape Shedu by mutating their UvsW helicase, which is involved in Holliday branch migration during repair and recombination events. These events include the formation of dsDNA linear intermediates, supporting the model that Shedu attacks linear DNA ends. Due to the diversity of the N-terminal domain across different Shedu systems, it is hypothesized that their N-terminal clamps also dictate specificity. *E. coli* Ppl is another single-gene ssDNA 3′-5′ exonuclease system that has a dual-requirement for activation: phage-mediated nucleotide pool depletion alleviates the inhibitory effect of NTPs bound to both Ppl’s PHP exonuclease and NTPase domains and the detection of 3’-OH ends generated by phage-encoded homing endonucleases. *P. aeruginosa* Gabija, as mentioned above, also interfaces at phage genome ends, monitoring the conflict between phage end-binding proteins and RecBCD [50]. The absence of RecBCD activity tells Gabija to prevent end-joining, leading to the inhibition of phage genome circularization. Structural analyses of Lamassu systems also suggest DNA-end binding as a detection mechanism [59].

Both Shedu and Gabija, do not normally target self-DNA, suggesting that both systems have additional specificity requirement(s) that prevent targeting of linear intermediates the host genome may form during replication errors, dsDNA breaks, or recombination. Heterologous expression of Shedu systems lead to cellular toxicity, suggesting that the N-terminal clamps, or perhaps other unidentified specificity determinants, may prevent species-specific self-targeting. Gabija can also target self when heterologously over-expressed or if RecBCD activity is ablated. RecBCD sequence diversity across prokaryotic species may be an explanation for this, but more studies are needed to explain how RecBCD activity regulates Gabija and how this differs across species.

## Membrane localization

DNA-targeting defense systems such as Zorya, Kiwa, and SNIPE include transmembrane embedded sensor proteins in addition to nucleases [60–63]. These systems require interactions between the transmembrane proteins and their nuclease effectors for phage defense, suggesting that spatial localization is a key specificity determinant. Type I Zorya consists of 4 proteins: ZorA, ZorB, ZorC, and ZorD. ZorA and ZorB form a proton-driven rotary motor complex embedded in the inner membrane [60]. It is hypothesized that membrane piercing by phage injection proteins pinch the peptidoglycan (PG) layer closer to the inner membrane, decreasing the PG-inner membrane distance to allow ZorB to bind to PG. ZorB-PG binding, coupled with increased ion flow through the transmembrane domain activates the ZorAB motor, which recruits the normally autoinhibited ZorC and ZorD to bind and degrade injecting phage genomes. Two-component Kiwa’s KwaA forms tetramers that are embedded in the inner membrane, each bound by four KwaB homodimers [62]. KwaB dimers act as anchors for these repetitive complexes to form lattices, cumulating into a KwaA-KwaB “fence-like” supercomplex. KwaA senses phage infection at the membrane and brings the supercomplex to the phage injection site, allowing KwaB to then bind to injecting DNA. KwaB binding prevents DNA replication and late gene transcription, but KwaB does not cleave DNA. The exact mechanism for phage infection sensing by KwaA is unknown. SNIPE is a membrane-bound nuclease that associates with a host-membrane-embedded protein complex, ManYZ, used by certain phages for genome injection. Upon infection, SNIPE is brought to the vicinity of the phage injection machinery where SNIPE’s cytoplasmic DUF4041 domain binds to the phage tape measure protein. This positions the nuclease domain to allow for cleavage of injecting phage DNA [63].

## DNA topology

In addition to phages, bacteria defend against other types of mobile genetic elements (MGEs) such as plasmids. Examples of such systems include the previously mentioned DdmDE pAgo system and SMC-like systems DdmABC (aka Lamassu) and Wadjet [22,64–66]. Both Wadjet and DdmABC consist of a structural maintenance of chromosome (SMC) family condensin-like complex in addition to a nuclease effector [64–66]. Once bound to plasmid or phage DNA, the SMC-like sensors are thought to either detect stalled replication forks at secondary DNA loop structures (DdmABC) or extrude circular DNA of smaller sizes until it encounters a “stuck” state (Wadjet). This then signals the activation or recruitment of the effector nucleases of these systems. Why these systems do not effectively cleave the host chromosome is still unknown, but proposed models include: distinct topology of the host chromosome due to plectonemic supercoiling (i.e., braid-like twisting), chromosomal size, or efficient DNA repair systems that counteract effector activities.

## Phage countermeasures

Phage survival is partly predicated on their acquisition and evolution of counter-defense measures (Table 1). Many phages incorporate their own unique base modifications into their genomes [37,67]. These modifications are not only involved in the regulation of operon expression, packaging phage genomes, and maintaining genome stability, but also protect viral DNA from host nucleases. For example, rather than cytosine, T4 phage DNA incorporates glucosyl-5-hydroxymethylcytosine (ghmC) bases which robustly protects T4 genomes from Gabija, Shedu, RM, type III Druantia, and qatABCD [68]. More than two-dozen modifications have been discovered across *Escherichia*, *Pseudomonas*, and *Salmonella* phages [37,67], suggesting that there may be other DNA modifications that interfere with defense systems, and unidentified nucleases that cleave DNA with these modifications. While base modifications exclude nucleases from accessing their substrate, even more elaborate mechanisms of achieving substrate protection have also been described. For example, jumbo phages such as phiKZ and phiKZ-like phages protect their genomes with a lipid-based vesicle and a protein-based nucleus-like compartment [69,70]. This mechanism shields the DNA from restriction and CRISPR-Cas enzymes and possibly other nucleases [71].

Phages may also express inhibitors of DNA-targeting systems. These include DNA mimics, proteins with negative surface charge that occupy the DNA binding sites of nucleases and compete against binding of viral DNA. In addition to some anti-CRISPRs (Acrs), T7 phage’s Ocr has been shown to inhibit RM and BREX [72,73]. Other inhibitors bind to or near oligomerization interfaces. In *V. cholerae*, OrbA directly binds BrxC of the BREX system, preventing multimerization of BrxC [74]. Gad1 binds around the Gabija complex forming a “cage” around the supercomplex [75,76]. How Gad1 binding inhibits Gabija activity is still unknown. T7 and *S. enterica* phage JSS1 deploy protein kinases as broad countermeasures that inhibit many DNA-targeting/binding proteins by phosphorylation [77,78] and T3 phage expresses a SAMase that allows it to avoid Type I RM by cleavage of S-adenosyl methionines (SAMs) [79]. Phages can also express adapter proteins, such as restriction alleviation (Ral) proteins, to utilize the host defense machinery to protect their own genomes. Ral interacts with RM modification subunits to enhance modification of phage genomes, thereby protecting their own DNA from restriction enzymes [80]. Druad1 also promotes site-specific methylation of phage genomes to evade type I Druantia [33]. Finally, some phages have their own repair and recombination systems that counter viral DNA cleavage [50,81–83]. A handful of other inhibitors of DNA-targeting systems have been discovered, such as broad defense inhibitors Bdi1 and Bdi2, Hachiman anti-defense Had-1, type I Zorya anti-defense ZadI-1, type-III Druantia anti-defense DadIII-1, old anti-defense Oad1, and Gabija anti-defense Gad2, but their mechanisms of inhibition are yet to be elucidated [76,84,85].

## Concluding remarks

The diversity in specificity mechanisms across DNA-targeting systems highlights the incredible complexity of the broader microbial immune repertoire and the many internal barriers phages must consider and overcome during infection. Much like CRISPR-Cas and RM, we expect the field to continue discovering new classes of DNA-targeting systems, compounding on the complexity of the DNA-targeting pool and, perhaps, defining new restriction mechanisms. In addition, bacterial genomes encode, on average, 5–10 known defense systems [8,17], which begs the question of how DNA-targeting systems may behave in the presence of other defense systems. Costa et al have shown evidence of synergistic effects between DNA-targeting system pairs, such as type II Zorya and type III Druantia, on phage restriction in *E. coli*, where anti-phage activity of any individual system is minimal, but significantly improves when the two systems are active [84]. Synergistic patterns likely differ between species due to different ecological environments, predatory agents, and bacterial life cycles, so similar studies across other bacteria will likely reveal new inter-defense compatibilities.

Despite significant progress in the discovery of DNA-targeting systems and their mechanisms for phage detection, major gaps in knowledge on how these systems restrict phage or plasmid DNA *in vivo* still need to be filled. In addition, more work is needed to understand the regulation of these systems in their native strains, as well as how host nucleoid-binding proteins may influence or regulate immune systems. A deeper understanding in these areas will greatly contribute to advancing efforts in phage therapeutics, comprehending microbial ecology and microbiome evolution, and biotechnological applications of DNA-targeting systems.
